# Supplementary material for: Splice-Junction-Based Mapping of Alternative Isoforms in the Human Proteome
Source: Cell Rep. Author manuscript; Available in PMC 2020 Jan 15. (PMC6961840; doi:10.1016/j.celrep.2019.11.026)
Supplement: 2 [file NIHMS1546469-supplement-2.zip › DF1/PXD006675/RightCoronaryArtery/RightCoronaryArtery_10_SLC2A11_SLEVIQSTEMEDELEPSLRPR.pdf]

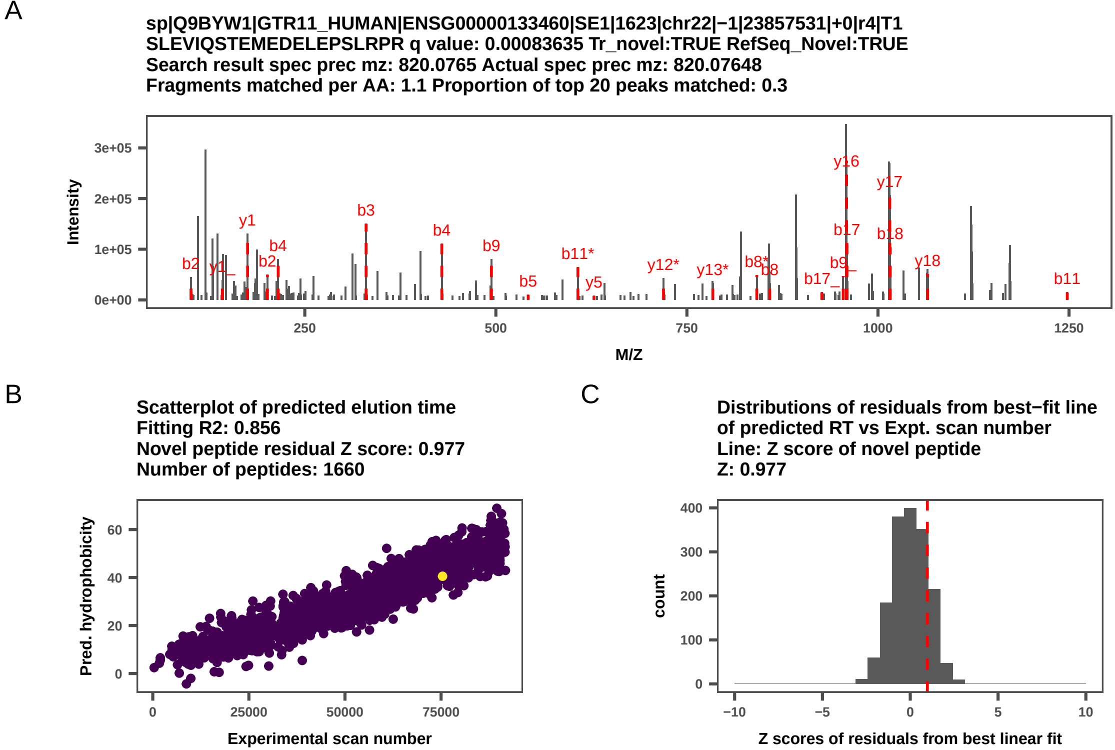

A
sp|Q9BYW1|GTR11_HUMAN|ENSG00000133460|SE1|1623|chr22|−1|23857531|+0|r4|T1
SLEVIQSTEMEDELEPSLRPR q value: 0.00083635 Tr_novel:TRUE RefSeq_Novel:TRUE
Search result spec prec mz: 820.0765 Actual spec prec mz: 820.07648
Fragments matched per AA: 1.1 Proportion of top 20 peaks matched: 0.3
Intensity
3e+05
2e+05
1e+05
0e+00
b2
y1_
y1
b2
b4
b3
b4
b9
b5
b11*
y5
y12*
y13*
b8*
b8
b17_
b9_
y16
b17
y17
b18
y18
b11
250
500
750
1000
1250
M/Z
B
Scatterplot of predicted elution time
Fitting R2: 0.856
Novel peptide residual Z score: 0.977
Number of peptides: 1660
Pred. hydrophobicity
60
40
20
0
0
25000
50000
75000
Experimental scan number
C
Distributions of residuals from best−fit line
of predicted RT vs Expt. scan number
Line: Z score of novel peptide
Z: 0.977
count
400
300
200
100
0
−10
−5
0
5
10
Z scores of residuals from best linear fit
